# Proximal versus distal bone transport for the management of large segmental tibial defect: a clinical case series

**DOI:** 10.1038/s41598-023-31098-6

**Published:** 2023-03-08

**Authors:** Yao Lu, Qian Wang, Cheng Ren, Ming Li, Zhong Li, Kun Zhang, Qiang Huang, Teng Ma

**Affiliations:** Department of Orthopedics, Hong Hui Hospital, Xi’an Jiaotong University, Xi’an, 710054 Shaanxi China

**Keywords:** Trauma, Fracture repair

## Abstract

This retrospective study compared proximal bone transport and distal bone transport in a series of cases diagnosed with large segmental tibial defects. Patients with a tibial segmental defect (> 5 cm) were eligible for inclusion. Twenty-nine patients were treated using proximal bone transport technique (PBT group) and 21 cases were managed by distal bone transport technique (DBT group). We recorded the demographic information, operation indexes, external fixation index (EFI), visual analog score (VAS), limb function scores, and complications. Patients were followed for 24–52 months. There was no significant difference in operation time, blood loss, time in frame, EFI and HSS score between the two groups (*p* > 0.05). However, the PBT group displayed better clinical effects than the DBT group, including higher AOFAS scores, lower VAS, and complication incidence (*p* < 0.05). In particular, the incidence of Grade-II pin-tract infection, transient loss of ankle movement, and foot drop was significantly lower in PBT group than that in DBT group (*p* < 0.05). Although both methods could be used safely for the management of large segmental tibial defects, the proximal bone transport may confer greater patient satisfaction because of better ankle functions and lower complications.

## Introduction

Large segmental tibial defects are common after high-energy injuries^1^. Infection after internal fixation may cause similar tibial injuries. They are problematic for patients, due to the long treatment course, repeated surgeries and possible loss of limb functions. This brings great physical and mental pressure to patients and their families. This is also a challenge for trauma surgeons, even if it is handled by experienced surgeons.

The main treatment methods for reconstruction of large segmental bone defects include Masquelet technique (also called induced membrane technique), fibular transplantation and Ilizarov bone transport^2–6^. Masquelet et al.^7,8^ introduced a two-stage method which involves radical debridement and insertion of antibiotic-loaded bone cement at the bone defect site for the first stage. The aim is to control infection and form induced membrane. An external fixator is used to maintain the stability of the injured limb. Bone reconstruction is performed in the second stage by removal of bone cement and filling with cancellous bones. In order to improve comfort, the external fixator can be replaced with an internal device, such as a nail or a plate. The advantages of Masquelet technique include simple operation and definite curative effects^2,3^. Yet, due to the obvious individual differences in the amount of autologous bones that can be obtained, this method is not applicable sometimes. Infection recurrence is disastrous for patients using this technique. Such patients will not only lose valuable implanted autogenous bones, but also be forced to undergo additional surgeries, and even face amputation. Vascularized fibular transplantation is another option to treat patients with long bone defects. This method requires good microsurgical skills^4,9^. However, the application of this technique is limited due to insufficient strength and re-fracture of the implanted fibula after operation.

**Figure 1:**
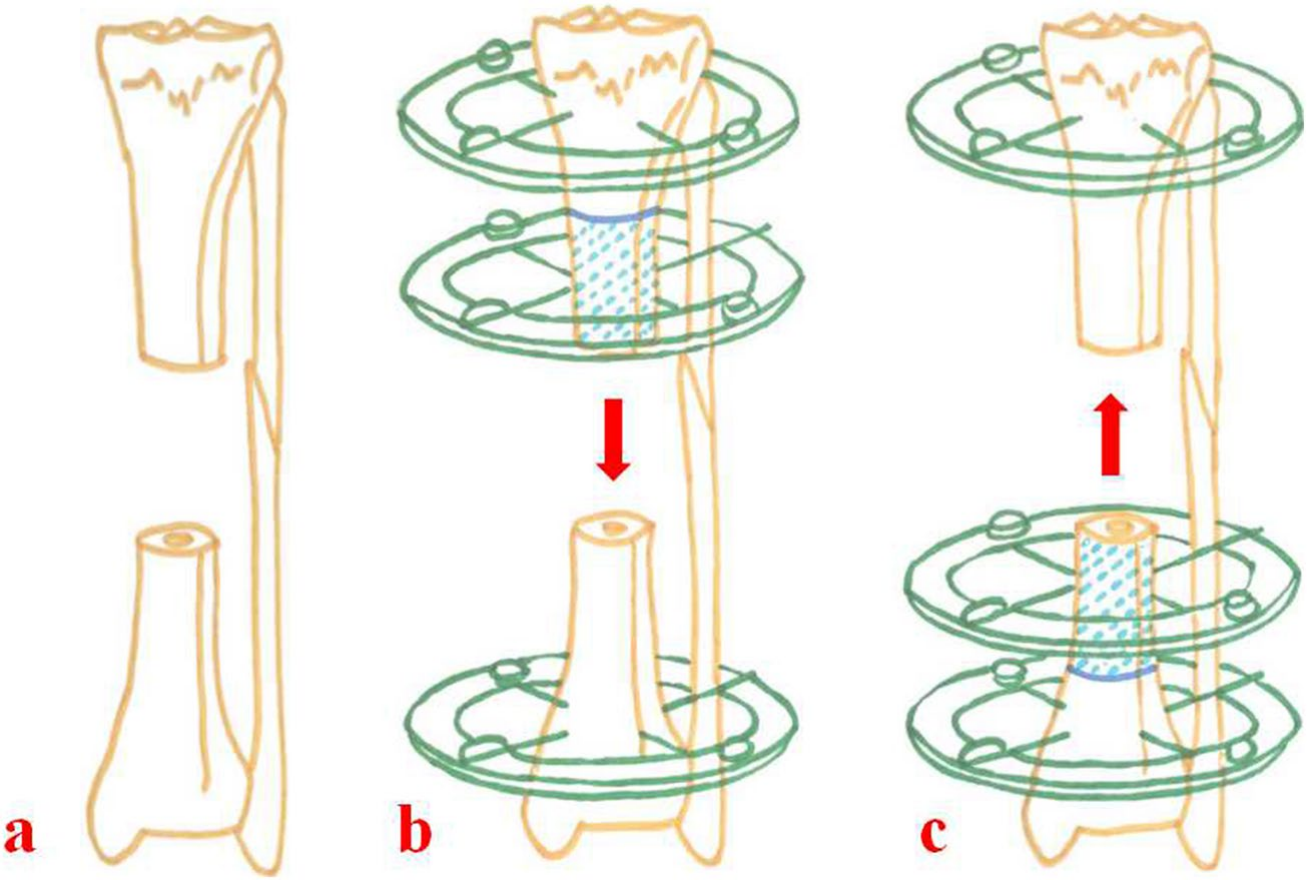
Schematic diagrams of proximal and distal bone transport. (**a**) Large bone defects of middle tibia. (**b**) Proximal osteotomy and bone transport technique. (**c**) Distal osteotomy and bone transport technique.

Ilizarov bone transport technique has been widely used as a limb salvage option for patients with segmental bone loss^3,5,6,9^. According to Ilizarov’s principle of tension-stress, the regenerative potential of bone tissues is stimulated by gradual and continuous tensile stress, and the gap generated by slow traction will be filled with new callus. It has the advantages of not only dealing with the problem of large bone loss, but also shortening, deformity, joint contractures, or soft tissue defects. In the process of distraction osteogenesis, nerves, vessels, tendons, and skin tissues are lengthened correspondingly. In order to shorten the external fixation time, several scholars have put forward different modified techniques, such as double-level bone transport, shortening and re-lengthening, bone transport over a nail or a plate, bone transport and then nailing^10–13^. These modified techniques expand the application scope of Ilizarov technique and increase patients’ satisfaction. In addition, based on our experience, when other bone reconstruction techniques fail, Ilizarov bone transport might be a final solution for patients with large segmental bone defects. However, there is a small problem that has not been clarified by previous studies. For patients with large tibial defects, whether proximal bone transport is superior to distal bone transport (as shown in Fig. 1) ? We focused on this topic and made a retrospective research. The aim of this study is to compare proximal bone transport with distal bone transport in a series of cases diagnosed with large segmental tibial defects and display our experience.

## Materials and methods

### Study design

The institutional review board (IRB) approval has been obtained from the ethics committee of Xi’an Hong Hui hospital. A written informed consent has been gotten from patients or their families. All methods were carried out based on relevant guidelines and regulations. Fifty cases with tibial segmental defects were collected from June 2011 to June 2019 in our institution. Twenty-nine patients were treated using PBT technique (the PBT group) while 21 cases were managed by DBT technique (the DBT group). Each patient was selected according to the following inclusion and exclusion criteria. The inclusion criteria encompassed the following points: (1) Patients over 18 years; (2) Patients were diagnosed with bone defects in the middle third of the tibia; (3) Patients with tibial defects longer than five centimeters; (4) Patients were treated by Ilizarov annular bone transport technique; (5) Patients with complete medical records. The exclusion criteria included five points: (1) Patients younger than 18 years; (2) Patients with major comorbidities and were unable to tolerate anaesthesia or surgery; (3) Patients were managed by other methods, not bone transport; (4) Patients with final amputation; (5) Patients with incomplete medical records or lost patients.

Based on each medical record in our institution, basic data of all patients were collected, including age, sex, bone loss, etiology, Gustilo-Anderson (GA) classification, body mass index (BMI) and follow-up duration. Acute trauma and osteomyelitis were the main causes for tibial segmental defects. Initial open injuries were classified according to GA classification. Bone loss was measured after radical debridement by the picture archiving and communication system (PACS). VAS of each patient was assessed at one month after operation. Time in frame and external fixation index were calculated when the transport fixator was removed. At the last follow-up, the Hospital for Special Surgery (HSS) score was used to evaluate knee functions, including pain (30 points), function (22 points), range of motion (18 points), muscle strength (10 points), knee flexion deformity (10 points), and stability (10 points)^14^. The American Orthopaedic Foot and Ankle Society (AOFAS) ankle-hindfoot score was used to assess ankle functions^15^. The total score is on a scale of 0 to 100, with 100 indicating no symptoms or impairments. For HSS and AOFAS scores, a higher score indicates better joint functions. Postoperative complications were recorded and divided into “problems” (treated nonoperatively), “obstacles” (treated operatively) or “sequelae” based on the principles proposed by Paley^16^. The clinical effects and functional recovery scales of the two groups were evaluated by trained and experienced surgeons.

### Surgical procedure

All patients were given a general examination after admission. The injured limb was routinely checked under the X-ray machine, and blood samples were obtained to monitor infection-related indexes. Bacterial culture and drug sensitivity test were taken regularly. Patients usually underwent staged surgeries. In the first stage, radical debridement was performed. All infected soft tissues, sequestrum, free bone fragments were completely removed. For patients with an open fracture, if the tissue vitality was doubtful, it was properly preserved. Debridement was carried out again as soon as possible to decide whether the doubtful tissues would be retained or not. For sequestrum or infected bones, segmental resection method was applied sometimes. Antibiotic-loaded Polymethylmethacrylate (PMMA) spacer was filled into the bone defect site. The injured limb was fixed by a temporary external fixator. Then, soft tissues were repaired according to the “reconstruction ladder” principle.

**Figure 2:**
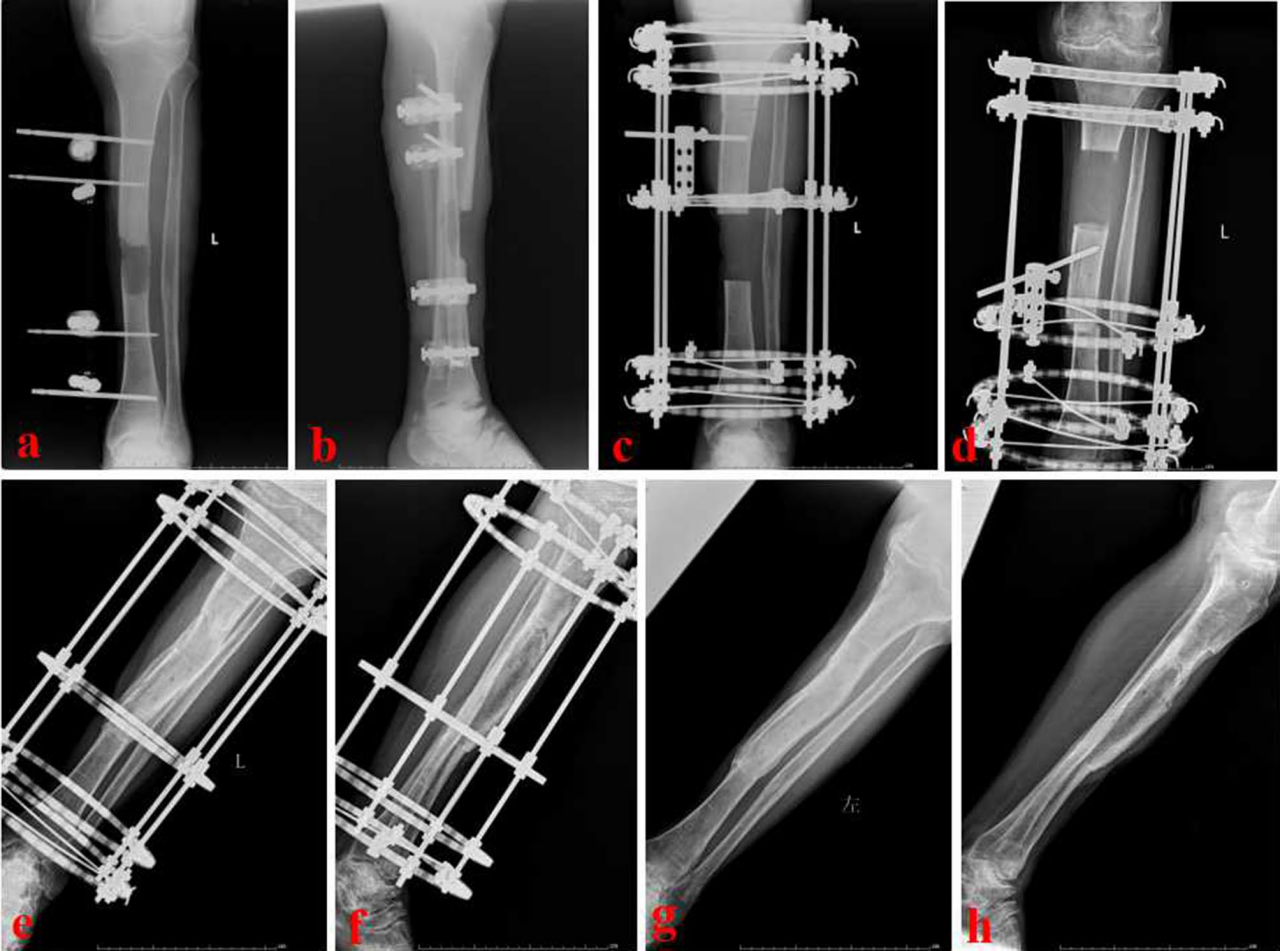
A 59-year-old male was successfully treated by the PBT technique. (**a**) and (**b**) The patient suffered from a severe infected tibial defects; (**c**) and (**f**) After radical debridement, the PBT technique was performed; (**g**) and (**h**): X-ray images showed that the bone defect was fully repaired. PBT: proximal bone transport.

**Figure 3:**
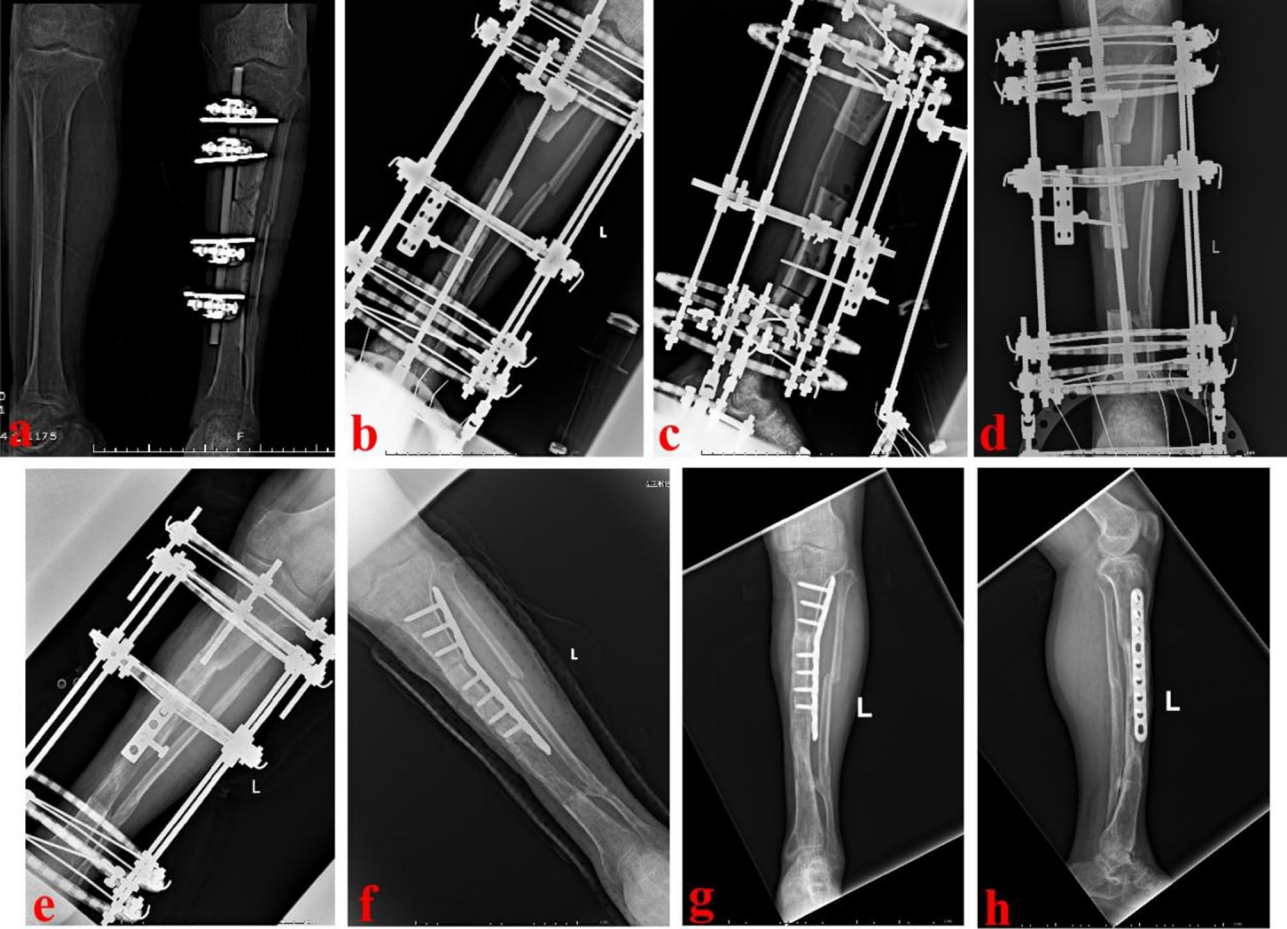
A 40-year-old male was treated by the DBT technique. (**a**) The patient suffered from osteomyelitis of middle tibia; (**b**) and (**c**): The DBT technique was performed after radical debridement; (**d**)–(**f**): Although the bone transport process successfully completed, docking site nonunion occurred and the patient was managed by bone grafting and internal fixation; (**g**) and (**h**): The docking site healed after bone grafting and internal fixation. DBT: distal bone transport.

When infection was completely controlled and the wounds healed, bone reconstruction was performed in the second stage. The temporary external fixator and PMMA spacer were removed. The injured lower leg was maintained in the center of the annular transport frame. Parallel to the knee and ankle joint surface, the transport frame was fixed by several Kirschner pins. According to the surgical plan, the distal or proximal osteotomy was performed. The transport segment was fixed by another two Kirschner pins. The alignment and rotation were checked by a C-arm image intensifier. After that, the wounds were washed and sutured. Drainage was inserted at the tibial defect site if necessary. One week after operation, the transport frame was adjusted for bone transport. The initial transport speed was one millimeter per day. After one month, the speed was adjusted according to the limb tolerance. Limb sensation and circulation were closely monitored during transport process. When the docking site was in contact, proper compression was given to promote the docking site healing. After the consolidation period finished and the docking site firmly healed, the transport frame was removed. Figure 2 showed a typical case of the proximal bone transport. On the contrary, Fig. 3 displayed a typical case of the distal bone transport.

Joint functional exercises were taken after operation. Patients were treated with anti-infammatory, detumescence, pain relief and other symptomatic treatments. Sensitive antibiotics were given for four to six weeks. According to bacterial culture results and drug sensitivity test, antibiotics were adjusted in time. Infection-related indexes were monitored regularly. X-ray films of the injured limb were rechecked every two weeks for the first two months. Patients were followed for at least two years through outpatient service, telephone, wechat platform, etc.

### Statistical analysis

Comparative statistical analyses between the PBT group and DBT group were performed in SPSS software 23.0 (IBM Company, Chicago, United States). Measurement data were expressed as mean ± standard deviation. Unpaired t test was used for comparisons between the two groups. Count data were analyzed using χ2 test. Statistical significance was set at *p* < 0.05.

## Results

The specific demographics of the two groups are shown in Table 1. There was no significant difference about demographic information for PBT and DBT patients (*p* < 0.05, Table 1).

**Table 1:** Demographics of the two groups.

Variable	PBT group (n = 29)	DBT group (n = 21)	*p* value
Age (years)	34 ± 9	36 ± 10	0.471
Sex (male/female)	18/11	14/7	0.738
Bone loss (cm)	7.8 ± 2.4	8.1 ± 2.6	0.680
Etiology			0.796
Acute trauma	14	10	
Osteomyelitis	9	8	
Others	6	3	
Gustilo-Anderson classification			0.999
GA I	4	3	
GA II	3	2	
GA IIIa	9	7	
GA IIIb	5	3	
GA IIIc	2	1	
Closed	6	5	
BMI (kg/m^2^)	25 ± 4	26 ± 4	0.388
Follow-up (months)	34 ± 7	35 ± 8	0.649

PBT stands for proximal bone transport. DBT stands for distal bone transport. GA stands for Gustilo-Anderson. BMI stands for body mass index.

As shown in Table 2, there was no significant difference for the mean operation time (*p* = 0.318), the mean blood loss (*p* = 0.126), the mean time in frame (*p* = 0.249), and the mean EFI (*p* = 0.088) between the two groups. However, for VAS at one month after operation, the points were lower in PBT group than that in DBT group (*p* < 0.001). For HSS score after removing the external frame, there was no significant difference for PBT and DBT patients (*p* = 0.137). AOFAS score was also recorded after removing the transport frame. The mean AOFAS score was higher in PBT group than that in DBT group, and the difference was significant (*p* < 0.001).

**Table 2:** Clinical and limb function evaluation of the two groups.

Variable	PBT group (n = 29)	DBT group (n = 21)	*p* value
Operation time (min)	135 ± 23	142 ± 25	0.318
Blood loss (ml)	53 ± 14	59 ± 13	0.126
Time in frame (months)	12.1 ± 3.7	13.3 ± 3.5	0.249
EFI (months/cm)	1.5 ± 0.2	1.6 ± 0.2	0.088
VAS score (points)	4.3 ± 0.7	5.2 ± 0.9	*p* < 0.001
HSS score (points)	82 ± 8	85 ± 6	0.137
AOFAS score (points)	85 ± 7	76 ± 9	*p* < 0.001

EFI stands for external fixation index. VAS stands for visual analog score. HSS stands for the Hospital for Special Surgery. ASFAS stands for the American Orthopaedic Foot and Ankle Society.

Complications encountered are shown in Table 3. According to the principles proposed by Paley, complications were divided into “problems” (treated nonoperatively), “obstacles” (treated operatively) or “sequelae”. In PBT group, eight problems occurred. Those suffered from Grade-II pin-tract infection were given local care and dressing change. Patients with transient loss of knee and ankle movement were guided to take joint functional exercises or accepted joint manual release. There were 20 obstacles happened in PBT group. Those with Grade-III pin-tract infection were given removal of the infected pins and inserting a new one. Patients with deep infection were given radical debridement again and sensitive antibiotics. Those with docking site nonunion were given autologous cancellous bone grafting, or with internal fixation. Patients with axial deviation were given surgical adjustment. The two suffering from re-fracture of the transport segment were given reduction and internal fixation. For those with soft tissue invagination, surgical release was performed regularly. The one with foot drop was given Achilles tendon lengthening and local soft tissue release. Four cases suffered from sequelae and rejected further corrections.

**Table 3:** Comparison of complications between the two groups.

Variable	PBT group (n = 29)	DBT group (n = 21)	*p* value
Problems			
Grade-II pin-tract infection	2(6.9%)	7(33.3%)	0.042
Transient loss of knee movement	5(17.2%)	2(9.5%)	0.716
Transient loss of ankle movement	1(3.4%)	6(28.6%)	0.035
Obstacles			
Grade-III pin-tract infection	4(13.8%)	3(14.3%)	0.716
Deep infection	2(6.9%)	1(4.8%)	0.772
Docking site nonunion	3(10.3%)	2(9.5%)	0.702
Axial deviation	4(13.8%)	2(9.5%)	0.986
Re-fracture of transport segment	2(6.9%)	1(4.8%)	0.772
Soft tissue invagination	4(13.8%)	1(4.8%)	0.567
Foot drop	1(3.4%)	6(28.6%)	0.035
Sequelae			
Others	4(13.8%)	1(4.8%)	0.567
Number of complications per patient	1.10 ± 0.49	1.52 ± 0.75	0.032

In DBT group, fifteen problems were observed. Besides, 16 obstacles occurred. Only one case encountered sequelae and rejected further surgeries. The treatment procedures of the complications in DBT group were similar to that of the PBT group. The incidence of Grade-II pin-tract infection, transient loss of ankle movement, and foot drop in PBT group was significantly lower than that in DBT group (*p* < 0.05). With respect to the mean number of complications per patient, patients in PBT group showed lower numbers than that in DBT group, and the difference was significant (*p* = 0.032).

## Discussions

Large segmental bone defects are a tough problem faced by trauma surgeons. As the soft tissues around the tibia are thin and weak, the tibia is conductive to bone defects after trauma^1^. Similarly, the incidence of osteomyelitis caused by infection after internal fixation is higher in the tibia than in the femur. At present, the goal of treatment is to repair bone defects, restore limb functions, prevent complications, and improve the life quality of patients. Different management options exist for addressing tibial defects, and the definite scheme is dictated by the personality and severity of the injuries, surgeons’ experience, patients’ preference, and implant availability. Common methods comprise autogenous bone grafting, vascularized free fibular transfer, forming an induced membrane and then bone grafting, or Ilizarov bone transport^2–9^. Amputation should also be considered for some cases involving substantial tibial bone loss and major comorbidities^17^.

Since the last century, Ilizarov technique has been widely used and has given surgeons an important option for treating patients with unequal limb length, infected non-unions with bone defects, angular, translational, or rotational deformities, joint contractures, and temporary fixation in patients with severely injured soft tissues^3,5,6,9^. This technique has obvious advantages in the treatment of patients with large segmental bone defects. It is performed percutaneously, and this will minimize soft tissue damage. The new callus generated during transport process, whether structure, functions, or thickness, is similar to the original bones. In addition, it allows early load-bearing and has nearly no limits on the size of the defects^18^. Bone transport technique has a high final success rate. Oh et al. showed that the final bone defect healing rate was more than 90% using bone transport method^19^. In single-level bone transport, when the bone defect site is located at the proximal tibia, osteotomy is performed at the distal part. Conversely, if patients suffer from distal tibial defects, proximal osteotomy will be carried out. For patients with bone defects in the middle third of the tibia, although proximal or distal osteotomy can both be performed, there are no studies comparing the effects of the two methods. Our study is the first report to compare the effects of proximal and distal bone transport.

Ankle problems are the most important reason of residual disability after successful application of the Ilizarov frame for the management of segmental tibial defects^20^. Though knee stiffness is largely improved with physiotherapy, foot and ankle stiffness persist and worsen despite bony union. This results in the final poor outcomes by using bone transport. Megas et al. observed stiffness of the ankle joint in 55% of patients and reported it as a common and severe residual problem after Ilizarov bone transport^21^. Li et al. retrospectively analyzed the clinical data of 40 patients with severe composite tibial and soft tissue defects who underwent free flap transplantation combined with Ilizarov bone transport^22^. Eight cases suffered from joint stiffness (ankle joint stiffness, foot drop, claw toe) in their study. We also observed that there was significant difference for ankle complications and ankle function scores between the proximal and distal bone transport. Based on our results, the PBT group showed higher AOFAS scores, lower incidence of transient loss of ankle movement, and foot drop than the DBT group. From the anatomical point of view, the proximal tibia is rich in muscle tissues, while at distal tibia it is mainly tendon tissues. When the transport pins pass through the muscle tissues, it has small influence on the adjacent joint movement. If the pins penetrate into the tendon tissues, the patient will reduce the movement of adjacent joints due to fear of pain. Therefore, distal bone transport has a greater impact on the ankle functions than proximal bone transport. Similarly, because of the cutting and pulling of the distal muscles and tendons by the transport pins, distal bone transport make patients feel more pain than the proximal method. This also explains why the PBT group got a lower VAS score at one month after operation, compared with the DBT group.

Pin-tract infection is especially reported as the most commonly seen complication in most studies of bone transport^23–26^. Out of 595 patients who were evaluated in a narrative review, with regard to information on pin-tract infection, 299 had pin-tract infection^20^. In our study, pin-tract infection was also the most common complication (16/50). Besides, the incidence of Grade-II pin-tract infection was significantly lower in PBT group than that in DBT group. This may be because the proximal tibia has more abundant soft tissues and better circulation than the distal tibia. Theoretically, since blood vessels penetrate the bone superiorly and run in a proximal–distal direction^27,28^, and so does the blood supply of the muscles and periosteum, the proximal bone transport will have a perfusion advantage. Adequate blood supply provides sufficient antibiotics and nutrients, and improves local anti-infection ability. Aktuglu et al. analyzed the studies using single-level bone transport technique in recent ten years. Their results showed that the mean complication rate per patient was 1.22^20^. In our study, the mean number of complications per patient was 1.10 ± 0.49 in PBT group and 1.52 ± 0.75 in DBT group. Importantly, the PBT group showed lower mean complication rate than the DBT group. Other scholars reported that the EFI ranged from 1.2 to 2.8 months/cm with a mean of 2.0 months/cm by using the single-level bone transport technique^29,30^. Our results of the mean EFI in the two groups (1.5 ± 0.2 months/cm vs. 1.6 ± 0.2 months/cm) were similar to the above studies.

This study has several limitations. As this study was a single-center study with limited cases, more rigorous and large-scale researches are needed. Because this was a retrospective case–control study, the chief surgeon would have personal preference for the patient’s treatment options. Even so, it would not obviously interfere the conclusions that we have gotten.

## Conclusion

Although both proximal and distal bone transport could be used safely for the management of large segmental tibial defects, the proximal bone transport may confer greater patient satisfaction because of better ankle functions and lower complications.

## Data Availability

The datasets analyzed during the current study are available from the corresponding author upon reasonable request.
